# Recent progress in three-terminal artificial synapses based on 2D materials: from mechanisms to applications

**DOI:** 10.1038/s41378-023-00487-2

**Published:** 2023-02-17

**Authors:** Fanqing Zhang, Chunyang Li, Zhongyi Li, Lixin Dong, Jing Zhao

**Affiliations:** 1 School of Mechatronical Engineering, Beijing Institute of Technology, 100081 Beijing, China; 2 Beijing Advanced Innovation Center for Intelligent Robots and Systems, Beijing Institute of Technology, 100081 Beijing, China; 3 Department of Biomedical Engineering, City University of Hong Kong, Kowloon Tong, 999077 Hong Kong, China

**Keywords:** Electronic devices, Electronic properties and materials

## Abstract

Synapses are essential for the transmission of neural signals. Synaptic plasticity allows for changes in synaptic strength, enabling the brain to learn from experience. With the rapid development of neuromorphic electronics, tremendous efforts have been devoted to designing and fabricating electronic devices that can mimic synapse operating modes. This growing interest in the field will provide unprecedented opportunities for new hardware architectures for artificial intelligence. In this review, we focus on research of three-terminal artificial synapses based on two-dimensional (2D) materials regulated by electrical, optical and mechanical stimulation. In addition, we systematically summarize artificial synapse applications in various sensory systems, including bioplastic bionics, logical transformation, associative learning, image recognition, and multimodal pattern recognition. Finally, the current challenges and future perspectives involving integration, power consumption and functionality are outlined.

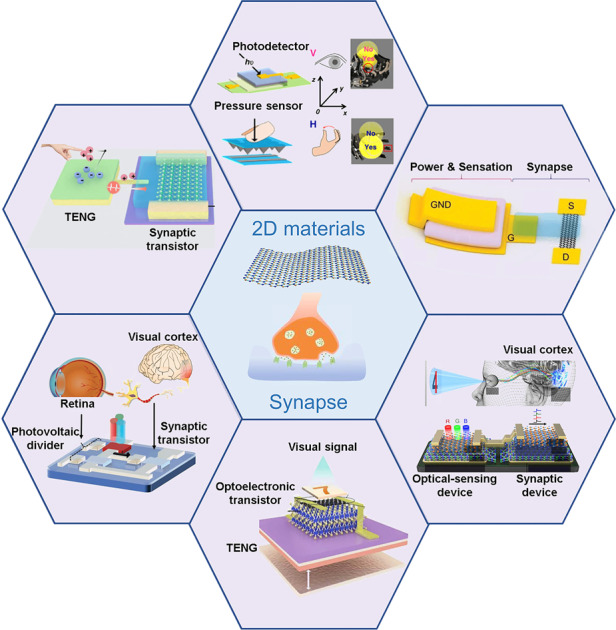

## Introduction

**Fig. 1:**
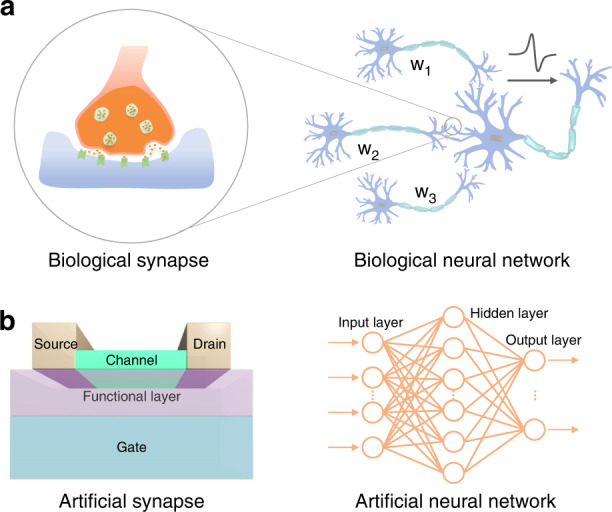
Comparison of artificial neural networks and biological neural networks. **a** Biological synapse and neural network (including three components: receptor, axon and synapse). **b** Artificial synapse and neural network (including three components: sensor, pathway, and memory)

Cognition and memory are the main sources of human intelligence. Therefore, human beings have vigorously developed intelligent electronics to imitate biological functions such as the multifunctional sensing and processing of neural signal transmission, storage and feedback. Even though the standard von Neumann architecture has given our software a literally free increase in performance by increasing the speed of every single component, the problem of a separate central processing unit and storage area limiting transmission speed remains a challenge^1–3^. In addition, high latency, excessive energy consumption and insufficient parallelism have become bottlenecks. In contrast, biological synapses control the plastic strength of connections between anterior and posterior neurons to realize data transmission^4^. Therefore, the neuromorphic system provides a novel efficient solution for processing large amounts of complex data. In this system, neuromorphic devices are applied to simulate synaptic plasticity, using electrical property changes to simulate the connection strength of biological synapses^5–50^. There are various structures of synaptic devices, including two-, three- and multiple-terminal structures, where the signal transmission path and weight modulation are different^51^. Typical two-terminal devices, such as memristors, phase-change memories, and atomic switches, can reach a small size and be integrated easily due to their simple structure^34,52–58^. In this architecture, the signal transmission and learning process perform asynchronously due to the lower number of terminals; thus, the signal is inhibited during the learning operation with the output signal as feedback to the synaptic device^59–64^. In comparison, three- and multiple-terminal synaptic transistors can not only realize signal transmission and self-learning processes simultaneously but also demonstrate high stability, repeatability and clear operating mechanisms^19,65–83^. Figure 1 shows a comparison between an artificial neural network and a biological network. At present, research on three- and multiple-terminal synaptic transistors is still preliminary, and neuromorphic transistors have mainly focused on four types: synapses based on floating gate-regulated, ferroelectric, photoelectric and electromechanical field effect transistors (FETs)^34,84–97^.

**Fig. 2:**
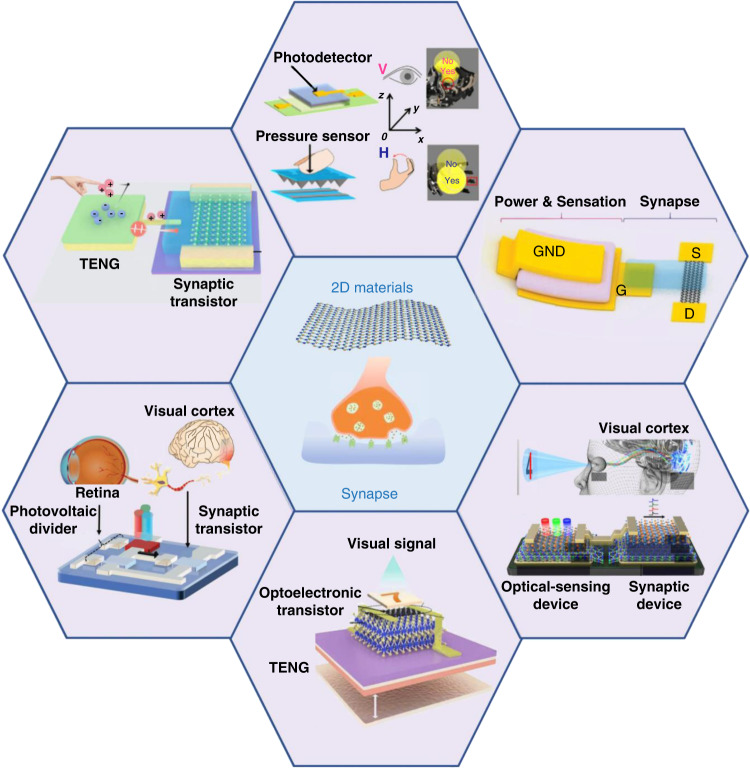
2D material artificial synapse and its advanced applications. Adapted with permission^113^. Copyright 2021, Springer Nature. Adapted with permission^114^. Copyright 2020, Springer Nature. Reproduced with permission^74^. Copyright 2019, John Wiley and Sons. Adapted with permission^30^. Copyright 2018, Springer Nature. Adapted with permission^115^. Copyright 2021, AAAS. Reproduced with permission^112^. Copyright 2019, John Wiley and Sons

Emerging 2D materials (such as graphene, transition metal chalcogenides (TMDs), and hexagonal boron nitride (h-BN)) have demonstrated excellent properties to realize various functions of biological synapses^35,36,76,77,93,94,98–105^. The atomic structures of these 2D materials provide high integration, suppressed short-channel effects and low leakage currents. Their unique 2D layered structures lead to large surface-to-volume ratios to sensitively perceive external stimulus signal changes. In addition, stacked 2D materials can act as channels with variable energy band structures^84,106–109^. In recent years, 2D material synaptic devices have attracted broad research interest (Fig. 2) for extensive applications. Their outstanding and stable mechanical, thermal, electrical, and optical properties enable them to serve as artificial synaptic devices with high stability and low-power consumption^16,26,30,36,38,73,74,110–128^.

In this article, we summarize recent research on artificial synapses based on 2D materials. First, we introduce various three-terminal synaptic devices with different operating principles, including the biomimetic principles of floating gate-regulated transistors, ferroelectric layer transistors, optoelectronic synaptic transistors, and electromechanical coupling transistors^32,33^. Second, we focus on the current development of artificial synapse properties excited by electrical, optical, mechanical, and hybrid stimulation. Third, we enumerate advanced applications of artificial synapses to mimic biological neural behaviors^129^. Finally, the challenges and future perspectives of artificial synapses are outlined.

## Three-terminal synaptic devices

Synaptic devices can be classified as two-, three- and multiple-terminal devices depending on the number of terminals. Typical devices with two terminals can be divided into magnetic random-access memories (MRAM), resistive random-access memories (RRAM), and phase-change memories (PCM)^130–132^. The electrical signal transmission and material resistance strength can be regulated by electrical signals between two electrodes.

**Fig. 3:**
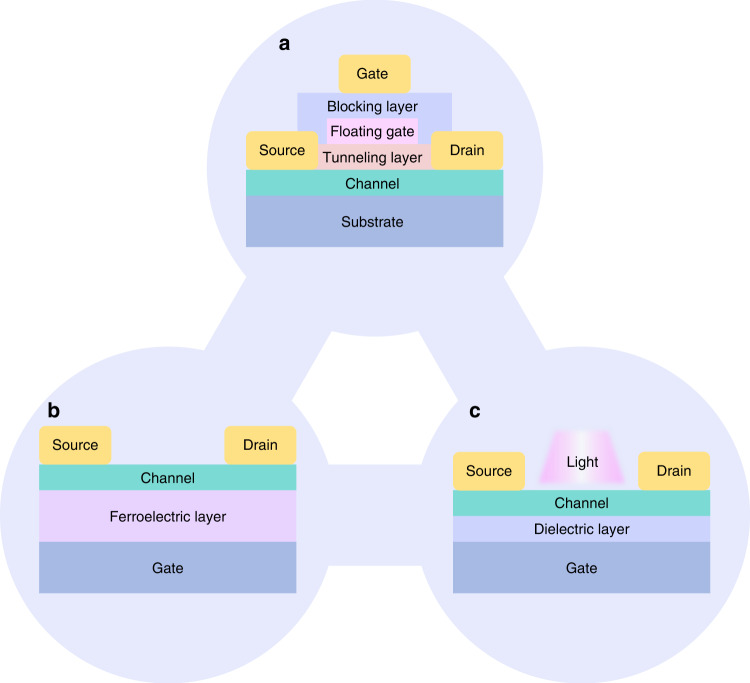
Structure diagrams of typical three-terminal devices. **a**–**c** Structures of the FGFET, FeFET, optoelectronic field effect transistor, and respectively

In contrast, triple-ended synaptic devices can obtain additional regulation provided by the third terminal. The synaptic property of the device can change by applying different voltages at the gate electrode. According to the various modulation mechanisms derived from different functionalized layers coupled with 2D materials, three-terminal artificial synaptic devices can be divided into synapses based on floating gate field effect transistors (FGFETs), ferroelectric field effect transistors (FeFETs), optoelectronic field effect transistors (OFETs), or electromechanical coupling field effect transistors (MFETs), as shown in Fig. 3^74,133–139^.

### Floating-gate synapses

The structure of an FGFET is shown in Fig. 3a. The floating gate layer inserted between the channel layer and gate electrode acts as a charge well, which is isolated by a tunneling and a blocking layer, respectively^140^.

The threshold voltage *V*_T_ of the FGFET is given by

$$V_T = {{{\mathrm{K}}}} - \frac{{Q_{{\rm{FG}}}}}{{C_{{\rm{CG}}}}}$$

where *Q*_FG_ and *C*_CG_ represent the floating gate charge and the capacitance between the control gate (CG) and floating gate (FG), respectively. *K* is a constant depending on device manufacturing processes.

When a writing gate voltage is applied, charges in the channel can cross over the tunneling layer through the Fowler–Nordheim (F–N) tunneling or channel hot electron injection mechanism and be stored in the floating gate layer. Thus, the threshold voltage of the device can be tuned by the *Q*_FG_ when a write/erase operation is performed^48,133,134,140–148^.

Paul et al. reported a molybdenum disulfide (MoS_2_)-based floating-gate synaptic device using graphene and hBN as the floating-gate and tunneling layers, respectively^133^. The device maintained an ideal subthreshold swing of 77 mV/decade under over four decades of drain current. In addition, the device, as a biological synapse, successfully simulated the weight update in response to external stimuli by modulating the channel conductance. The high durability (>10^5 ^s) and low energy consumption (~5 fJ for a single pulse) of the device laid the foundation for FGFET synapses in neuromorphic applications.

Compared with conventional dielectric layers, high-k materials are better at suppressing the short-channel effect during device downscaling and can improve the performance of synaptic devices based on FGFETs. For example, Joon Young Kwak and coworkers optimized the thickness of the blocking layer (HfO_2_) and the tunneling layer (Al_2_O_3_) in a MoS_2_/graphene structure to achieve linear synaptic weight updates^149^. The device exhibited spike-timing-dependent plasticity (STDP) behavior and demonstrated the possibility of constructing a synaptic device using mass-grown 2D materials, implying a route for spiking neural network (SNN) neuromorphic hardware.

### Ferroelectric synapses

An FeFET has advantages of fast operating speed, long retention time, large on/off ratios and low-power consumption, making it promising for use in artificial electronic synapses. Ferroelectric materials are low-symmetry crystals, and the specific polarization direction can be formatted under an appropriate external electric field. Due to the nonlinear relationship between the polarization of the ferroelectric material and the applied electric field, a significant electrical hysteresis can be observed in the polarization–electric field (P-E) loops^150–154^. Therefore, the polarization state of the ferroelectric layer can be changed by applying an appropriate gate pulse voltage, thus allowing the synapse to be tuned by multidomain polarization switching. In addition, the ferroelectric material can also act as the dielectric layer. Therefore, the gate voltage can modulate the polarization state in real time and influence the channel carrier density, leading to a nonvolatile storage state^92,135,136,155–159^.

Tian et al. integrated an organic ferroelectric thin film P(VDF-TrFE) as a dielectric layer in a MoS_2_ FET and obtained a switching ratio of ~10^4^^160^. Typical biological synaptic plasticity, such as long-term potentiation (LTP), long-term depression (LTD), and STDP, was successfully simulated by the dynamic resistive switching of the device. Due to the polarization flipping ability of the organic ferroelectric material, the device demonstrated low energy consumption (~1 fJ per pulse) and decade-long durability.

In addition, Tang et al. constructed a synaptic transistor using an α-In_2_Se_3_ 2D ferroelectric semiconductor as the channel material^161^. The conductance of the α-In_2_Se_3_ channel was adjusted by the gate voltage pulse, resulting in a synaptic weight change. The authors simulated basic synaptic behaviors such as single-, paired- and multiple-spike responses by adjusting the gate voltage pulse width. This approach used ferroelectric materials as channel layers and provided a new path for device miniaturization compared with a device using ferroelectric material as the dielectric layer. Wang et al. added more control terminals to the α-In_2_Se_3_ ferroelectric transistor, realizing nonvolatile storage and implementing neuromorphic computing^162^. The memory window can reach 6 V and remain stable even after 500 cycles. In addition, the device showed a fast response time and could switch on/off under 40 ns write pulses. In addition, the flexible synaptic plasticity of the device with ~fJ power consumption showed little influence on the nonvolatile memory performance when synaptic weights were updated^163^.

### Optoelectronic synapses

Optical detection, processing and memory are fundamental requirements for an artificial vision system. The image capture system was separated from the memory unit for conventional devices. The optoelectronic synapse can realize optical information perception and storage within a single device, enabling the synergistic processing of optical and electrical signals^138,164–168^. Optoelectronic synapses can simulate synaptic behavior with high energy efficiency, strong robustness, and good parallelism to simulate functions as retinal neurons in human eyes^103,137,169,170^.

According to a previous study, 2D materials were widely used in photoelectric devices because of their rich energy band structure. Excitons can be produced under optical illumination and be moved inductively by the electric field between the source and drain electrodes. The carrier density in the channel affected the threshold voltage of the device, which exhibited light-tunable synaptic plasticity, as shown in Fig. 3c. Therefore, researchers have sought to achieve continuous, reversible and nonvolatile responses based on photoelectric synapses, leading to visual neural computing applications.

Seo et al. constructed an optical nerve device based on the h-BN/WSe_2_ structure and realized both synaptic and optical sensing functions^30^. The conductivity of the WSe_2_ channel was adjusted by the number of captured electrons in the oxygen plasma-treated h-BN weight control layer. The near-linear weight update performance provided stable conduction states with less than 1% variation. Additionally, the device showed low-power consumption with only 66 fJ under a single spike operation at 0.3 V.

Moreover, Sun et al. constructed synapses tuned by both optical and electrical stimulation based on the MoS_2_/h-BN structure^171^. The ionization and neutralization of intrinsic defects in h-BN can be co-stimulated by both optical and electrical spikes. Therefore, the synaptic weight was enhanced and suppressed, enabling unique bidirectional weight updating. The high accessibility (<1% change between cycles), long retention (>21 days), highly dynamic conductance range (>384) and moderate asymmetry (<3.9) of the device provided a maximum accuracy of 96.1% in human electrocardiogram recognition.

### Electromechanical synapses

Synaptic devices tuned by mechanical stimuli have been extensively investigated because they integrate both external mechanical stimuli perception and subsequent signal processing^74,139,172–174^. Therefore, the electromechanical synapse provides a new route for future applications in artificial electronic skin and neuromorphic interfaces for robotic and human interactions^80,175–178^.

Chen et al. presented the first piezoelectric artificial sensory synapse based on a piezoelectric nanogenerator (PENG) coupled with an ionic gel gated graphene FET^74^. The piezoelectric output induced by the mechanical strain tuned the FET property, enabling the tactile signal input and transmission process. At the same time, the weight of the artificial synapse could be effectively modulated by strain pulses, realizing potentiation/inhibition, spike-time-dependent plasticity and pair-pulse facilitation. The synapse provided a new way to construct artificial nerves with efficient perceptual and neuromorphic computing capabilities.

Yang et al. proposed a multifunctional artificial synapse with a coupling MoS_2_ FGFET and triboelectric nanogenerator (TENG) for mechanical plasticity^179^. The synaptic weight was modulated by mechanically changing the TENG displacement. The frictional potential coming from the TENG was coupled to the MoS_2_ FGFET, modulating the postsynaptic current and affecting the synaptic weights. The authors successfully mimicked classic enhanced and inhibited synaptic plasticity using different active interactions. Additionally, the device could implement simple logic operations simultaneously, making it a favorable candidate for building mechanically derived artificial neural networks and providing a possible route to perform neuromorphic logic switching and data storage simultaneously.

In addition, our group prepared nanographene/MoS_2_ floating-gate memory coupled with a TENG to realize multilevel storage states^75^. The device can be triggered by both mechanical and optical stimuli without applying an additional gate voltage. During the programming process, both the mechanical motion of the TENG and the incident light can drive stored electrons in the nanographene layer to the MoS_2_ channel. In contrast, the reverse motion of the TENG can penetrate electrons back to the charge trapping layer, leading to an erasure process. Due to the excellent mechanical properties of the 2D material, the device, integrated on a flexible PET substrate, exhibited stable (10^5 ^s) storage performance with a programmed erasure ratio up to 10^7^ even under strains greater than 1%. This electromechanical device paved the way for the development of next-generation low-power bionic synaptic systems with instant human–computer interactions.

## Plasticity of artificial synapses

The human brain shows better computing capabilities than supercomputers, including ultrafast response speed and low-power consumption due to high-density signal processing. There are ~10^11^ neurons in the human brain, and every neuron is connected with another ~10^4^ neurons, thus forming a highly interconnected and complex network with large-scale parallel computing functions. Biological synapses play an important role in information transmission in the nervous system and have time-dependent plasticity. Synaptic weight can be used to describe the strength of the connection between two neurons, which is achieved by adjusting the ion concentration (such as Ca^2+^, Na^+^, K^+^, etc.). As a result, learning, memory and computing functions can be realized by changing the weights^180–190^. Simulating the plastic characteristics of synapses can replicate the basic principles of the nervous system. The corresponding biomimetic synapses based on electronic devices can mimic biological synaptic behavior, forming an important branch of neuromorphic electronics and injecting new vitality into artificial intelligence development. Here, we discuss the plasticity of artificial synapses, including STP (excitatory postsynaptic current (EPSC), inhibitory postsynaptic current (IPSC), paired pulse facilitation (PPF) and paired pulse depression (PPD)) and LTP (STDP). Synaptic plasticity can be divided into two main types: STP and LTP, depending on the time of activity change^163,191–193^. By enhancing or inhibiting cerebral cortex activity in a short time between milliseconds and minutes, STP conducts synaptic transmission and realizes spatiotemporal neural activity. In contrast, LTP is widely recognized as the biological basis for long-term learning and memory^194–197^.

### STP

#### EPSCs and IPSCs

A synapse is characterized by a presynaptic pouch-like structure whose interior is composed of synaptic vesicles and mitochondria. The adjacent presynaptic membrane faces the postsynaptic membrane. Impulse signals, one of the most common signal transmission forms, play an important role in neural activity. When the human body is stimulated by an external stimulus, the corresponding impulse signal then forms and is transmitted to the presynaptic membrane. After receiving the pulse signal, the neurotransmitter of the synapse is released and transmitted to the postsynaptic membrane, which generates the membrane potential. EPSCs and IPSCs are the most fundamental neural activities that process complex information. To understand the physical mechanism of IPSCs and EPSCs in artificial synaptic devices under electrical modulation, the effect of relative positive/negative control gate voltage pulses on channel currents was analyzed^196,198,199^. The source–drain current of the synaptic transistor remained stable over time before the presynaptic pulse was applied. Depending on the relative positive or negative voltage pulse, the source–drain current was enhanced (EPSC) or suppressed (IPSC), respectively. It is worth noting that the current cannot return monotonically to the initial level even after the pulse is removed. Conversely, after the positive (negative) voltage pulse jumps to the initial level, the source–drain current acts as a postsynaptic current, exhibiting a slow relaxation phenomenon.

**Fig. 4:**
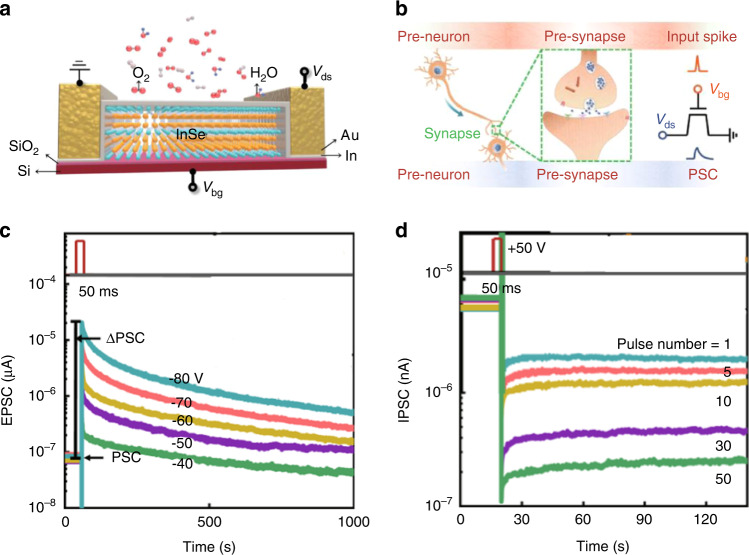
Structure schematic and emulation of the synaptic functions of InSe FETs. **a** Schematic diagram of the InSe FET exposed to ambient conditions. **b** Schematic illustration of a biological synapse and the InSe artificial synaptic device. **c** EPSC generated by applying several input spikes with different voltage amplitudes under the enhanced condition. **d** Plot of IPSC changes over 120 s after stimulation by various numbers of input pulses. Adapted with permission^202^. Copyright 2020, Springer Nature

In the post-Moore era, on-chip growth integration faces great challenges. 2D van der Waals materials have attracted extensive interest in next-generation nanoelectronics due to an atomic-scale thickness that is easily integrated^200,201^. In addition, some van der Waals materials, such as black phosphorus (BP), indium selenide (InSe), mercury sulfide (HfS_2_), molybdenum telluride (MoTe_2_), and layered organic materials (Ruddlesden‒Popper perovskite), are naturally sensitive to oxidative effects and tunable charge trapping. Correspondingly, transistors are believed to realize synaptic characteristics. Yang et al. reported an oxidation-enhanced van der Waals InSe artificial synapse, which successfully mimicked the basic bidirectional neuromorphic behavior of EPSCs and IPSCs^202^. InSe possesses a small effective electron mass and good intrinsic charge transport properties. In particular, the unstable characteristic of the air and large surface area to volume ratio led to hysteretic behavior. The changed microstructure of InSe FETs under ambient conditions confirmed that the native oxide formed at the bottom of the InSe channel can act as a unique charge-trapping layer to tune the charge transport behavior. This oxidation-induced InSe artificial synaptic device and corresponding image recognition system, based on an ANN, realized basic synaptic functions. The schematic structure of the artificial synapse based on surface-doped InSe FETs is shown in Fig. 4a. The applied gate voltage pulse as the input spike triggering the PSC can be compared to the biological synapse working process (Fig. 4b). The peaks of EPSCs were gradually augmented with increasing pulse voltage, corresponding to excitatory synaptic behavior (Fig. 4c). The value of the voltage spikes can be greatly reduced for low energy consumption by decreasing the thickness of the dielectric layers. Furthermore, Fig. 6d shows the typical IPSCs extracted as a function of retention time by varying the number of input spikes, illustrating the simulation of LTP behavior. In addition, the electrical properties of the device gave the way to simulate the flexible plasticity of PPF and STDP. This universal research method can be applied to MoTe_2_, HfS_2_, BP and other ultrasensitive van der Waals materials, which are sensitively affected by oxygen doping, opening up opportunities to build efficient neuromorphic computing systems.

#### PPF and PPD

PPF is a common manifestation of short-range synaptic plasticity in nervous system processing. It describes the phenomenon of spike-inducing EPSC enhancement when the second spike follows the previous spike immediately, leading to a presynaptic calcium ion concentration increase and in turn triggering synaptic vesicles to release large amounts of neurotransmitters. In contrast, PPD is a short-range depression, which is considered a type of negative feedback in the nervous system and is often attributed to the depletion of released vesicles. Synaptic inhibition plays an important role in processing perceptual adaptation and sound localization and enhancing information transfer efficiency^203–205^.

**Fig. 5:**
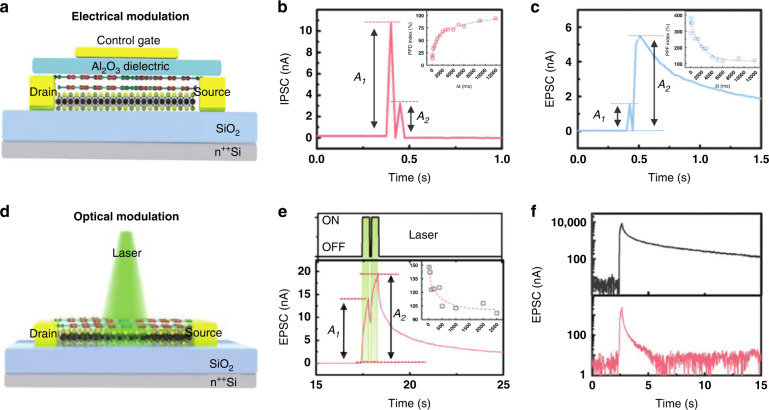
Artificial synaptic PPF behaviors modulated by electricity or light. **a** Schematic architecture of the MoS_2_/PTCDA hybrid heterojunction synaptic transistor under electrical modulation mode. **b** IPSC of the device triggered by a pair of *V*_cg_ pulses. **c** EPSC of the device triggered by a pair of *V*_cg_ pulses. **d** Schematic structure of the optical device. **e** EPSC of the device triggered by a pair of laser pulses. **f** STP to LTP transition by *V*_bg_ modulation. Reproduced with permission^210^. Copyright 2019, John Wiley and Sons

Recently, TMDs such as MoS_2_ and WSe_2_, with unique interfacial structural, electrical and optical properties, have been reported as promising candidates for complex neuromorphic applications. Moreover, 2D organic materials such as perylene-3,4,9,10-tetracarboxylic dianhydride (PTCDA) have received increasing attention not only because of their excellent optoelectronic properties but also because of their excellent compatibility with most inorganic 2D materials. Wang et al. demonstrated a novel 2D MoS_2_/PTCDA heterojunction synaptic transistor, which exhibited good optoelectronic modulation and biomimetic synaptic plasticity^206^. As shown in Fig. 5, the device can mimic biological synaptic behavior through both electrical and optical modulation. Transferred electrons at the MoS_2_/PTCDA heterojunction interface led to the corresponding STP and LTP synaptic behavior, similar to the neurotransmitter release process in biological synapses. Electrical and optical spikes and the heterojunction channel current corresponded to presynaptic input spikes and PSCs in biosynapses, respectively. The synaptic device can successfully simulate PPF and PPD behaviors when a pair of relatively positive or negative V_cg_ pulses are applied to the gate (Fig. 5b, c). Because both MoS_2_ and PTCDA can strongly absorb green light, a 532 nm laser pulse was used to replace the gate voltage spike without fabricating the top gate, as shown in Fig. 5d. When the laser irradiates the hybrid semiconductor, activated electrons can transfer from PTCDA to MoS_2_, leading to a proliferated PSC. The EPSC meant electrons gradually returned to the PTCDA after withdrawing the laser pulse; therefore, the PSC returned to the original level. In addition, increasing the interval between two laser pulses resulted in a decrease in the PPF, which was consistent with the result under electrical modulation (Fig. 5e). The applied positive back gate voltage can convert STP to LTP (Fig. 5f) due to electrons returning from MoS_2_ to PTCDA, prolonging the recovery relaxation time. The device relied on carrier transfer that occurs at the hybrid heterojunction interface and successfully achieved dynamic filtering and long-term weight changes.

### LTP

#### STDP

STDP, an application for Hebbian learning rules in the nervous system, adjusts the weight of intersynaptic connections by setting the timing of pre- and postsynaptic pulse sequences in neurons. This phenomenon was also considered one of the basic principles of brain learning and memory^25,207–209^.

**Fig. 6:**
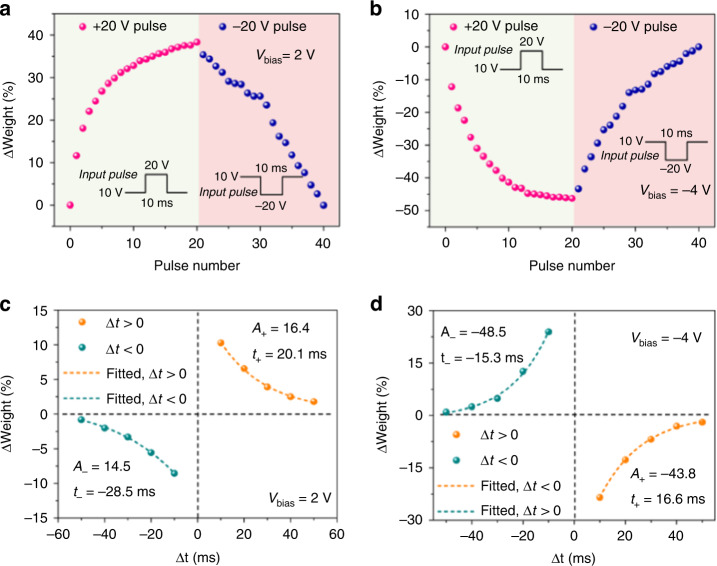
Artificial synaptic device based on a vdW junction. Excitatory synapse (**a**) and inhibitory synapse (**b**). (**c**) and (**d**) are the STDP characteristics under the same bias coming from (**a**) and (**b**), respectively. Adapted with permission^29^. Copyright 2017, American Chemical Society

Conventional artificial synapses based on memristors or transistors can achieve simple synaptic functions. However, it lacked the ability to dynamically reconfigure excitatory and inhibitory responses without adding modulation terminals. Tian et al. constructed a tunable heterojunction artificial synapse structure using BP and tin selenide (SnSe), simulating the biological synaptic effect of releasing excitatory and inhibitory neurotransmitters simultaneously^29^. Heterosynaptic devices typically rely on a third active terminal to modulate synaptic responses. The heterojunction device mimicked synaptic characteristics such as potentiation, inhibition, and STDP (Fig. 6a–d). The junction between the mid-bandgap material BP and SnSe can lead to tunable rectifying electrical properties, which is analogous to the single axon-dendritic synaptic connection process, providing a reconfigurable synaptic signature between excitatory and inhibitory responses^29^.

**Fig. 7:**
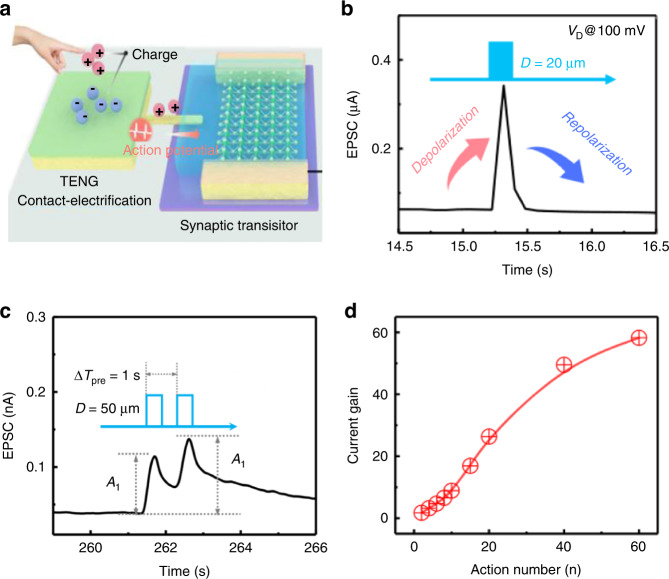
Contact-electrification-activated artificial afferents. **a** Schematic illustration of the artificial afferent. **b** EPSC responses under one CS action. **c** The typical PPF response under paired CS actions. **d** The current gain (the ratio of *A*_n_/*A*_1_) vs. action number. Adapted with permission^113^. Copyright 2021, Springer Nature

TENGs are capable of efficiently converting mechanical energy from the surrounding environment into electrical power. The coupling effect between triboelectric potential and semiconductor transport properties can potentially be used to mimic the function of biological sensory neurons or afferent nerves. Inspired by the phenomena in which bioreceptors capture touch signals to generate postsynaptic action potentials, Yu et al. fabricated an artificial afferent nerve activated by a contact mode. The energy dissipation of contact-electrification (CE)-activated artificial afferent nerves has been significantly reduced to the femtojoule level (11.9 fJ per spike)^113^. Tribo-potential modulation caused by the contact charged gate activation of synaptic transistors generated postsynaptic action potentials, as shown in Fig. 7a. The TENG was coupled to a MoS_2_-based ion gel-regulated transistor. The potential induced anion/cation migration to form EDLs in the ionic gel, changing the Fermi level of MoS_2_ channels and effectively triggering EPSCs. Stimulated by continuous and paired contact–separation (CS) mechanical actions, self-activating artificial afferents represented typical PPF behavior, in which a second spike elicited an increase in EPSCs (Fig. 7c). The tunability of the PPF index initialized by paired CS actions suggested that CE-activated artificial afferents had excellent short-term synaptic plasticity. In addition, the artificial afferent can also further enhance plasticity under multiple consecutive CS action pulses. This behavior is similar to saturated neurotransmitters under multiple presynaptic regulation. The current gain was defined as A_n_/A_1_ and was closely related to the number of CS actions (Fig. 7d), demonstrating that the plasticity of artificial synapses was gradually enhanced with increased stimulation times. A device using EDL gate control can successfully demonstrate spatiotemporal touch pattern recognition on flexible substrates. This work represents a promising strategy for developing next-generation biomimetic electronics, low-power neuromorphic devices, directly interacting electronic prosthetics and even neurorobots.

## Advanced applications of artificial synapses

Single synaptic devices have realized various synaptic features. Building a complete neural network system has become one of the most important branches of neuroelectronics. Multimodal synaptic devices based on 2D materials can simulate the learning and memory process of the human brain by synergizing various perceptions such as vision, smell and hearing. For example, emerging opto-mechanical synapses broke through the limitation of traditional electrical synaptic devices and provided a diverse route to change the synaptic weight. The emerging artificial synapse with synergistic multimodal plasticity can be applied to hybrid-modal neuromorphic chips and unconventional convolutional neural networks, which can be used in artificial retinas and intelligent robot applications^5,210–213^.

**Fig. 8:**
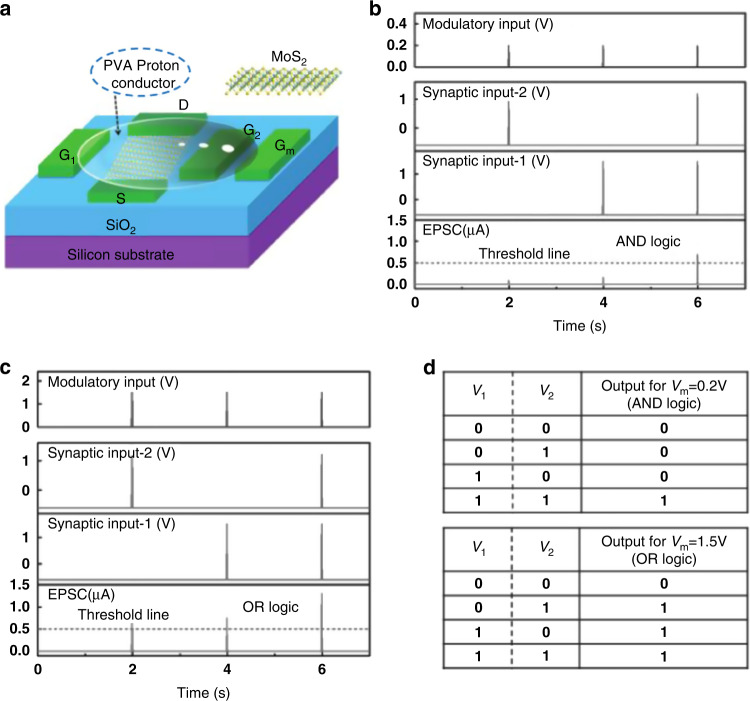
Multi-gate neuromorphic transistor for logic operations. **a** Schematic of a multiple-gate neuromorphic transistor based on MoS_2_. **b**, **c** represent the EPSC response caused by combining two presynaptic driving inputs with a regulated input, in which the voltage is 0.2 V in (**b**) and 1.5 V in (**c**). **d** Truth tables. Reproduced with permission.218 Copyright 2017, John Wiley and Sons

In recent years, three-terminal devices have been proposed as artificial neuromorphic synapses that can mimic the typical functions of biological synapses, such as dynamic logical circuits, self-learning, and STDP^186,214,215^. In 2017, Jiang et al. fabricated a multiterminal MoS_2_ neuromorphic transistor that successfully simulated EPSCs, PPF and spike logic regulation^216^. By applying multiple presynaptic inputs, pulse-dependent logical operations, multiplicative neural coding and spatiotemporally neuronal gain modulation can be simulated in the neuromorphic device. Figure 8a shows the integration function of multiple presynaptic dendrites, in which G_1_ and G_2_ represent each presynaptic-driven input terminal. Gm represents the presynaptic modulation terminal. The authors realized the logic functions of “AND” and “OR” by adding two voltage pulses, as shown in Fig. 8b–d.

**Fig. 9:**
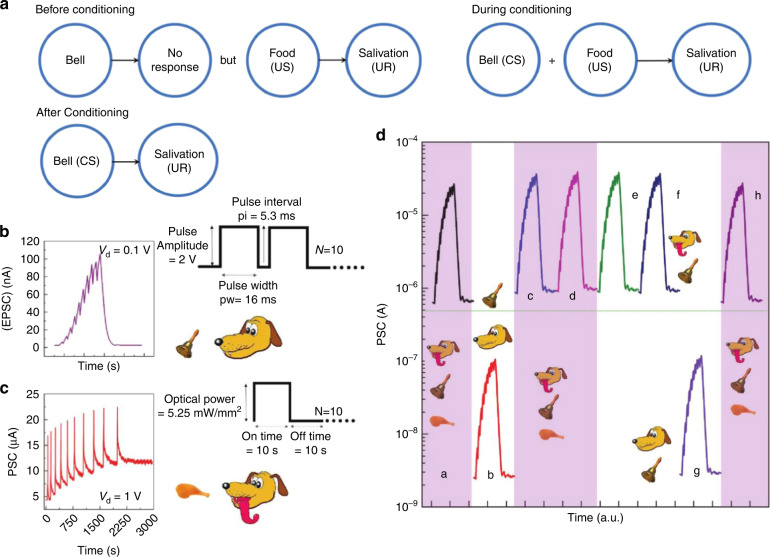
Pavlovian associative learning. **a** Schematic diagram of Pavlov’s dog experiment. **b** Electrical pulses as conditioned stimulus (CS) with EPSC < 500 nA (no salivation). **c** Optical pulses as unconditioned stimulus (US) with EPSC > 500 nA (salivation). **d** Imitation of Pavlovian conditioning. Reproduced with permission^27^. Copyright 2018, John Wiley and Sons

Conditional release is a typical form of associative learning in biology. John et al. reported a MoS_2_ three-terminal neuromorphic transistor that can mimic classic Pavlovian conditioning^27^. Researchers have established an association between STDP and the classic conditional reflex. The electrical pulses mimicked the process of ringing the bell (Fig. 9b), while the light pulses mimicked the feeding behavior (Fig. 9c) due to the different storage capacities for light and electrical pulses. After 40 cycles of training by light (US) and electrical pulse stimulation (CS), an efficient correlation between the two stimulations was established. In addition, Pavlovian conditioning can be performed under all-light stimulation. Furthermore, the 2D MoS_2_ neuromorphic transistor demonstrates comprehensive synaptic behavior for the first time, exhibiting different electronic, ion electronic, and photosensitivity operation modes. This optoelectronic synapse has an ultrafast propagation speed without interconnection problems, which shows outstanding application prospects in intelligent optical neurocomputing systems.

**Fig. 10:**
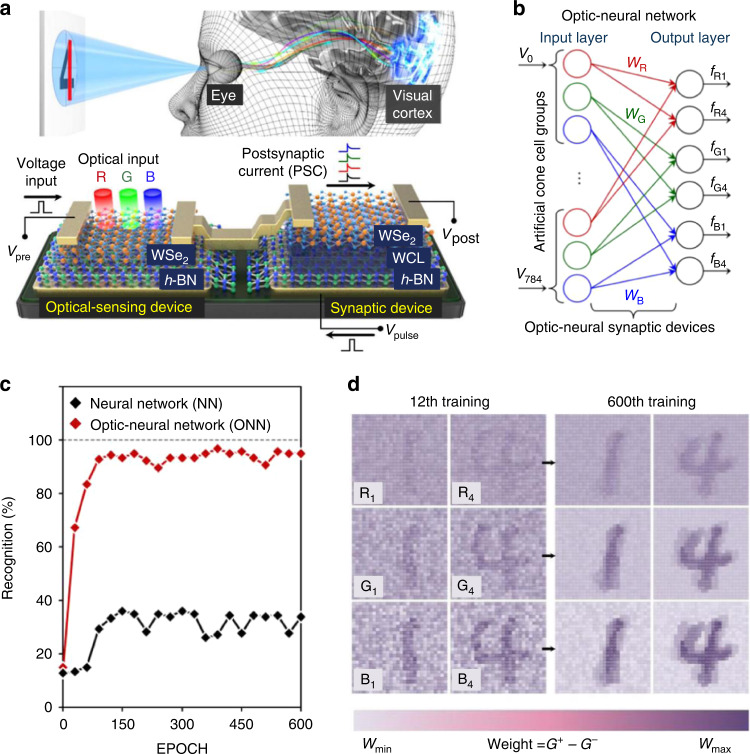
Artificial optical neural network with color recognition function. **a** Schematic of nerves in the human visual system versus the ONS device. **b** Developed ONN for recognition of 28 × 28 RGB-colored images. **c** Recognition rate as a function of the number of training epochs. **d** Weight mapping images after the 12th and 600th training epochs. Adapted with permission^30^. Copyright 2018, Springer Nature

Since Mead conducted the first experiment to simulate the brain’s biological neural network (BNN) in the 1980s, researchers have successfully simulated BNNs with various synaptic devices. Furthermore, Seo et al. proposed an optical-neural synapse (ONS) device based on the h-BN/WSe_2_ heterojunction, providing a possible route to integrate sensing and training functions for complex pattern recognition tasks^30^. The device can simulate the human visual system’s color and color mixed mode with recognition capabilities. In optical-neural networks, synaptic devices exhibit near-linear weight update trajectories, providing stable conduction states for color and color-mixing pattern recognition. The synaptic device used O_2_ plasma-treated h-BN as the charge trapping layer, as shown in Fig. 10a. An optical-neural network (ONN) was constructed based on an ONS device (Fig. 10b), which showed a corresponding response to light with different wavelengths and demonstrated better recognition results (exceeded 90%) than a traditional neural network (below 40%) (Fig. 10c). Therefore, the synaptic weight values are reproduced and visualized as the training number increases in Fig. 10d.

**Fig. 11:**
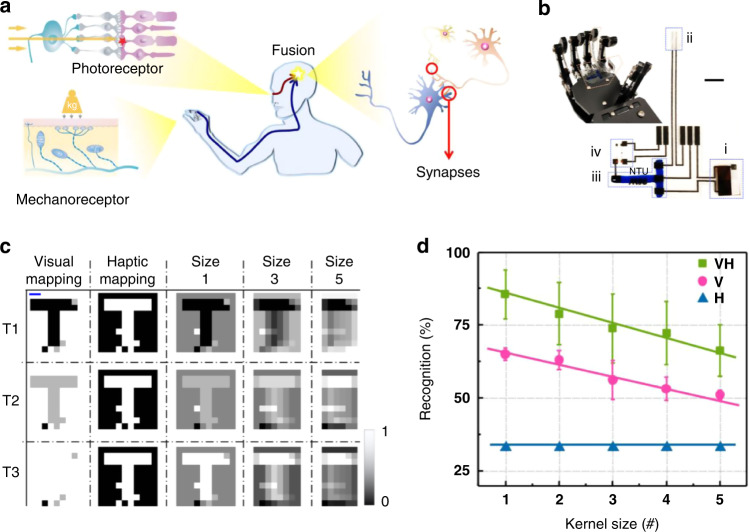
An artificial sensory neuron with visual-haptic fusion. **a** Visual-haptic fusion by a biological neural network. **b** The modified BASE patch on the robotic hand and the magnified image of the BASE patch. **c** The sensing data of each pattern obtained from the VH fusion matrix are fed into a perceptron for recognition. **d** The normalized mapping results based on unimodal information (visual or haptic) and VH fusion information. Adapted with permission^114^. Copyright 2020, Springer Nature

Humans have complex neural network systems that can analyze environmental information in parallel with multisensory cues. Wan et al. developed a bimodal artificial sensory neuron to implement a sensory fusion process that collected visual and pressure information through photodetectors and pressure sensors, respectively^114^. The bimodal information was transmitted through ionic transistors. Sensory neurons were activated by both visual and tactile stimuli, demonstrating enhanced task recognition ability after fusion. As shown in Fig. 11b, c, by combining manipulator pressure and LED light signals on the ball, researchers designed a matrix with visual-haptic fusion. which can fully extract shape and transparency information. This work simulated tactile and visual sensory fusion at the neuronal level, helping to build a highly integrated perception system to improve current robotics and artificial intelligence.

## Conclusion and outlook

This review summarizes the research progress of electrical, optical and mechanical responsive three-terminal artificial synapses based on 2D materials and their applications in artificial sensory systems, including biomimetic plasticity, logical transformation, associative learning, image recognition and multimodal pattern recognition. In recent years, novel three-terminal artificial synapses have successfully mimicked basic synaptic functional behaviors and exhibited gate tunability. Tri-terminal neuromorphic transistors have the advantage of processing data in parallel with multiple presynaptic inputs and simulating spatiotemporal dynamic logic. Neural computing based on three-terminal devices can play a more important role in pattern recognition and decision-making applications relying on massively parallel, highly interconnected neural circuits, good fault tolerance, self-learning ability and ultralow power consumption compared with traditional von Neumann architectures.

2D materials with excellent physical and chemical properties provide a potential platform to realize artificial synapses and neural networks. The reliability of the 2D channel material is a key factor affecting the operation of synaptic devices. van der Waals heterostructure artificial synapses can tune synaptic weights, which depend on the band alignment of vertically stacked materials. However, the difficulty in massively scalable and repeatable fabrication and defects introduced by heterostructures led to device performance variation. Therefore, high-quality wafer-scale 2D material synthesis promoted the development of practical human perception systems and neuromorphic computing.

A previous study initially focused on the electrical stimulation of artificial synapses. However, mechanical and photonic synapses have attracted more attention because modulations can link tactile and visual interactions. In particular, optical stimulation enabled the wireless transmission of synaptic devices. Considering that biological neurons have extremely high efficiency and ultralow power consumption, in the future, ultrasensitive, low-power, multimodal modulated and high-density integrated devices will become key requirements for building brain-like electronic systems. Furthermore, the integration and interconnection of neuromorphic devices are formidable challenges. Therefore, the reduced device size will help to increase the 3D integration density and reduce the power consumption. It is also necessary to process larger-sized wafers. In addition, matrixed neuromorphic transistors integrated with various sensors, which are compatible with conventional complementary metal oxide semiconductor (CMOS) technology, can realize more comprehensive and intelligent artificial sensory systems (e.g., audio, vision, touch, smell). Predictably, the artificial sensory system can be used in wide applications, including wearable devices, intelligent robots, and prosthetics. Considering the rapid development of materials science, computer science, artificial intelligence, medical care and other fields, artificial neural networks and synaptic electronics will achieve faster development.
